# Detection of Genome Donor Species of Neglected Tetraploid Crop *Vigna reflexo-pilosa* (Créole Bean), and Genetic Structure of Diploid Species Based on Newly Developed EST-SSR Markers from Azuki Bean (*Vigna angularis*)

**DOI:** 10.1371/journal.pone.0104990

**Published:** 2014-08-25

**Authors:** Sompong Chankaew, Takehisa Isemura, Sachiko Isobe, Akito Kaga, Norihiko Tomooka, Prakit Somta, Hideki Hirakawa, Kenta Shirasawa, Duncan A. Vaughan, Peerasak Srinives

**Affiliations:** 1 Program in Plant Breeding, Faculty of Agriculture at Kamphaeng Saen, Kasetsart University, Kamphaeng Saen, Nakhon Pathom, Thailand; 2 Genetic Resources Center, National Institute of Agrobiological Sciences, Tsukuba, Ibaraki, Japan; 3 Kazusa DNA Research Institute, Kisarazu, Chiba, Japan; 4 Department of Agronomy, Faculty of Agriculture at Kamphaeng Saen, Kasetsart University, Kamphaeng Saen, Nakhon Pathom, Thailand; National Institute of Plant Genome Research, India

## Abstract

*Vigna reflexo-pilosa*, which includes a neglected crop, is the only one tetraploid species in genus *Vigna*. The ancestral species that make up this allotetraploid species have not conclusively been identified, although previous studies suggested that a donor genome of *V. reflexo-pilosa* is *V. trinervia*. In this study, 1,429 azuki bean EST-SSR markers were developed of which 38 EST-SSR primer pairs that amplified one product in diploid species and two discrete products in tetraploid species were selected to analyze 268 accessions from eight taxa of seven Asian *Vigna* species including *V. reflexo-pilosa* var. *glabra*, *V. reflexo-pilosa* var. *reflexo-pilosa*, *V. exilis*, *V. hirtella, V. minima*, *V. radiata* var. *sublobata*, *V. tenuicaulis* and *V. trinervia* to identify genome donor of *V. reflexo-pilosa*. Since both diploid and tetraploid species were analyzed and each SSR primer pair detected two loci in the tetraploid species, we separated genomes of the tetraploid species into two different diploid types, viz. A and B. In total, 445 alleles were detected by 38 EST-SSR markers. The highest gene diversity was observed in *V. hirtella*. By assigning the discrete PCR products of *V. reflexo-pilosa* into two distinguished genomes, we were able to identify the two genome donor parents of créole bean. Phylogenetic and principal coordinate analyses suggested that *V. hirtella* is a species complex and may be composed of at least three distinct taxa. Both analyses also clearly demonstrated that *V. trinervia* and one taxon of *V. hirtella* are the genome donors of *V. reflexo-pilosa.* Gene diversity indicates that the evolution rate of EST-SSRs on genome B of créole bean might be faster than that on genome A. Species relationship among the *Vigna* species in relation to genetic data, morphology and geographical distribution are presented.

## Introduction

The Leguminosae genus *Vigna* comprises about 100 species. These species are morphologically diverse and geographically widespread and mainly found in Africa (African *Vigna*; subgenus *Vigna*) and Asia (Asian *Vigna*; subgenus *Ceratotropis*) [1]. The Asian *Vigna* comprises 21 species in which seven are domesticated and/or cultivated in various geographical and climatic regions, and cropping systems in Asia [2]. The seven domesticated/cultivated species include *V. aconitifolia* (moth bean), *V. angularis* (azuki bean), *V. mungo* (black gram), *V. radiata* (mungbean), *V. reflexo-pilosa* (créole bean), *V. stipulacea* (jungli bean) and *V. umbellata* (rice bean). All Asian *Vigna* species are diploid having 11 haploid chromosomes (2n = 2x = 22) with the exception for créole bean which is a tetraploid species with number of haploid chromosome of 22 (2n = 4x = 44). In fact, créole bean is the only natural amphidiploid in the subtribe Phaseolinae [3]. Cytogenetic analyses of *V. reflexo-pilosa* showed that the species formed 22 bivalents without multivalent at meiosis and was, therefor considered to be an amphidiploid [4] [5].

Cultivated and wild forms of créole bean are classified as *V. reflexo-pilosa* var. *glabra* and *V. reflexo-pilosa* var. *reflexo-pilosa*, respectively [6]. Wild créole bean is widely distributed in East, Southeast and South Asia, and across the islands from the west to the north Pacific islands. It is also found in Papua New Guinea and northern Australia [7] [8] [9]. The cultivated créole bean was formerly recognized as a glabrous variety of mungbean, *V. radiata* var. *glabra* [1]. Then, it was treated as a distinct species, *V. glabrescens* [3]. It is differentiated from its wild progenitor principally by thick glabrous stem and erect growth habit and reported to be cultivated as pulse in Vietnam and the Philippines or as forage in India, Mauritius and Tanzania [2]. This crop shows resistance to several insect pests and diseases such as bruchids, bean fly, powdery mildew, and cucumber mosaic virus [10], and is partially cross-compatible with mungbean [11]. Thus, créole bean has potential to be a gene source for breeding other *Vigna* crops. In addition, cultivated créole bean could be considered as a novel crop for the future and wild créole bean as a wider genepool to improve cultivated créole bean.

The origin and genome donors of créole bean have been the subject of debate. Based on the study on isozyme banding patterns and interspecific hybridization among the *Ceratotropis* species, Egawa et al [10] [12] proposed that *V. reflexo-pilosa* var. *reflexo-pilosa* is the descendant of natural interspecific hybridization between *V. trinervia* and *V. minima* followed by spontaneous chromosome doubling. An accession used as *V. minima* in Egawa et al. [10] [12] was correctly identified as *V. hirtella* by Konarev et al. [13], and was used in the present study (No. 21, JP108851, from Malaysia). Proteinase inhibitors polymorphism study in Asian *Vigna* by Konarev et al. [13] supported that one genome donor of créole bean is *V. trinervia* and suggested that the other genome donor is most likely *V. hirtella* or its closely related species. The phylogenetic studies based on plastid sequence data also supported that *V. trinervia* is a genome donor of créole bean [14] [15]. In contrast, rDNA-ITS sequence variation showed high similarity between créole bean and *V. exilis*, *V. hirtella* and *V. umbellata* [16], suggesting that one of these species is the genome donor of *V. reflexo-pilosa.*

The objective of this study was to determine the putative genome donor species of tetraploid créole bean using EST-SSR markers. To do so, we developed EST-SSR from azuki bean and used them to analyze accessions of créole bean and other *Vigna* species that are candidate genome donor species.

## Materials and Methods

### Plant Materials

**Figure 1:**
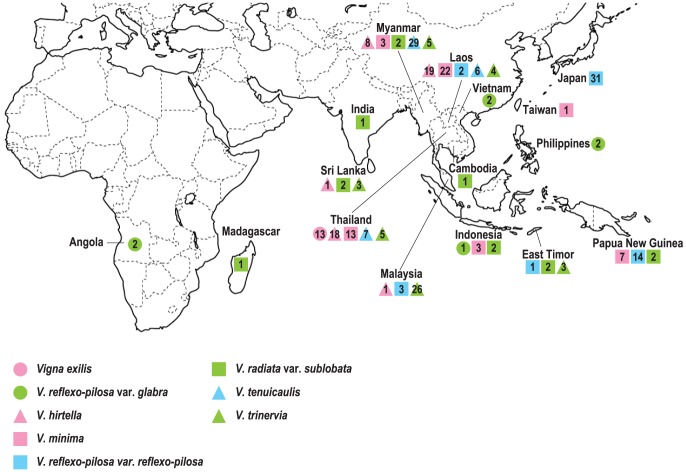
Distribution of 286 accessions of 8 taxa of the genus *Vigna* subgenus *Ceratotropis*. Numbers of accessions analyzed are shown within circles, triangles and squares.

A Japanese azuki bean cultivar ‘Erimo-shouzu’ (*V. angularis* var. *angularis*, accession no. JP37752) obtained from the Genebank, National Institute of Agrobiological Sciences (NIAS), Tsukuba, Japan was used for development of EST-SSR markers. Eight accessions consisting of four major *Vigna* crop species; azuki bean, rice bean, black gram and mungbean were used for assessing transferability and polymorphisms of the EST-SSR markers (Table 1). Two hundred and sixty-eight accessions from eight taxa of seven Asian *Vigna* species (Figure 1, Table S1) including 7 of *V. reflexo-pilosa* var. *glabra*, 51 of *V. reflexo-pilosa* var. *reflexo-pilosa*, 13 of *V. exilis*, 47 of *V. hirtella*, 49 of *V. minima*, 13 of *V. radiata* var. *sublobata*, 42 of *V. tenuicaulis*, and 46 of *V. trinervia* were used to analyze the genome origin of *V. reflexo-pilosa*.

**Table 1:** A summary of transferability and polymorphism of 1,429 azuki bean EST-SSR markers in the four Asian *Vigna* species.

Code	Species	Domestication status	Common name	Genebank acc. no	Origin	Linkage map	PCR products amplified	%	Simple banding pattern	%	Complex banding pattern	Exceeded of expected size	Number of markers compared between cultivated and wild	Polymorphic between cultivated and wild	%
VAC	*V. angularis* var. *angularis*	Cultivated	Azuki bean	JP81481	Japan	Han et al. (2005)	1327	92.9	1100	82.9	46	181	1307	236	18.1
VAW	*V. nepalensis*	Wild		JP107881	Nepal	Han et al. (2005)	1312	91.8	1090	83.1	45	177			
VMC	*V. mungo* var. *mungo*	Cultivated	Black gram	JP219132	Thailand	Chaitieng et al. (2006)	1216	85.1	1007	82.8	39	170	1196	164	13.7
VMW	*V. mungo* var. *silvestris*	Wild		JP107873	India	Chaitieng et al. (2006)	1194	83.6	998	83.6	36	160			
VUC	*V. umbellata*	Cultivated	Rice bean	JP217439	Myanmar	Isemura et al. (2010)	1324	92.7	1091	82.4	48	185	1296	187	14.4
VUW	*V. umbellata*	Wild		JP210639	Thailand	Isemura et al. (2010)	1304	91.3	1073	82.3	48	183			
VRC	*V. radiata* var. *radiata*	Cultivated	Mungbean	JP229096	Thailand	Isemura et al. (2012)	1197	83.8	987	82.5	44	166	1198	277	23.1
VRW	*V. radiata* var. *sublobata*	Wild		JP211874	Myanmar	Isemura et al. (2012)	1204	84.3	985	81.8	42	177			

### Development of Azuki Bean EST-SSR Markers

Total RNA was extracted from seedlings and young pods of Erimo-shouzu using Plant RNA Purification Reagent (Invitrogen, CA, USA). Purification of polyadenylated RNA and conversion to cDNA were performed as described by [17]. Synthesized cDNA was resolved by 1% agarose gel electrophoresis, and fragments ranging from 1 to 3 kb were recovered. The recovered fragments were cloned into the *Eco* RI-*Xho* I site of the pBluescript II SK- plasmid vector (Stratagene, CA, USA) and introduced into the *E. coli* ElectroTen-Blue strain (Stratagene, CA, USA) by electroporation. To generate ESTs, plasmid DNAs were amplified from the colonies using TempliPhi (GE Healthcare UK Ltd, Buckinghamshire, England) and subjected to sequencing using a BigDye Terminator Cycle Sequencing Ready Reaction Kit (Applied Biosystems, CA, USA). The reaction mixtures were run on an automated ABI PRISM 3730 DNA Analyzer (Applied Biosystems).

Sequencing chromatograms were converted into nucleotide bases with Phred [18] [19], and the sequences derived from the vector and linkers were removed with CROSSMATCH [19]. The EST reads were quality-trimmed with TRIM2 [20] using the Phred quality score ≥20, and ambiguous regions including more than ten X or N bases were trimmed. Contiguous, high-quality reads ≥100 bp were submitted to the DDBJ/EMBL/GenBank databases under the accession numbers HX939204 to HX950377 (11,174 entries). The PHRAP program with default parameters was used to cluster and identify non-redundant azuki bean ESTs [19].

Simple sequence repeats (SSRs) ≥15 nucleotides in length, which contained all possible combinations of di-nucleotide (NN), tri-nucleotide (NNN), and tetra-nucleotide (NNNN) repeats, were identified from the non-redundant azuki bean ESTs using fuzznuc program in EMBOSS [21] for SSRs within two mismatches. Primer pairs for amplification of SSR-containing regions were designed based on the flanking sequences of each SSR with the aid of the Primer3 [22] so that the amplified fragment sizes were between 90 bp and 300 bp in length. The newly developed markers were designated as VES (Vigna EST-derived SSR) markers.

### EST-SSR Marker Analysis

Total genomic DNA was extracted from young leaves of each *Vigna* accession using the method described by [23] with a slight modification. The DNA was quantified against a lambda DNA on 1.0% agarose gel stained with ethidium bromide and diluted to 5 ng/µl for PCR amplification.

Amplification, transferability and polymorphisms of all the EST-SSR markers were confirmed using eight accessions, two each from azuki bean complex, blackgram, rice bean and mungbean (Table 1). Five microliters of PCR reaction mixture including 0.5 ng of total genomic DNA, 0.02 U BIOTAQ DNA polymerase (BIOLINE, UK), 1x PCR buffer (BIOLINE, UK), 3 mM MgCl_2_, 0.2 mM dNTPs and 2 pmol of the forward and reverse EST-SSR primers. The PCR thermal cycling was performed as follows: 94°C for 1 min; 3 cycles of 94°C for 30 s and 70°C for 30 s, followed by 3 rounds of the same program in which the annealing and extension temperatures were decreased by 2°C every 3 cycles; 3 cycles of 94°C for 30 s, 62°C for 30 s and 72°C for 30 s, followed by 2 rounds of the same program in which the annealing temperatures were decreased by 2°C every 2 cycles; 30 cycles of 94°C for 30 s, 55°C for 30 s and 72°C for 30 s, and a final cycle at 72°C for 10 min. The PCR products were separated by 10% polyacrylamide gel electrophoresis in tris-borate-ethylene diamine tetraacetic acid (TBE) buffer according to the standard protocol, and banding patterns in the gel were stored as pictures. The characteristics of PCR products such as intensity, banding pattern and size range were recorded for each accession and EST-SSR markers. Polymorphism between wild and cultivated accessions in each species was recorded.

Out of 1,429 azuki bean EST-SSR markers, 175 markers with good transferability in all the four major *Vigna* crop species were labeled with fluorescent dyes and further examined for polymorphism in a panel of eight taxa of Asian *Vigna* consisting of 16 accessions, two accessions for each species randomly chosen from the 268 accessions (Table S1). The 5′-end of the reverse primers were labeled with one of the following fluorescent dyes; 6-FAM (blue), HEX (green), and NED (yellow) (Applied Biosystems). Five microliters of PCR reaction mixture contained 5 ng of genomic DNA, 1× QIAGEN Multiplex PCR Master Mix, and 5 pmol of the forward and reverse primers. PCR reactions were performed in a GeneAmp PCR System 9700 (Applied Biosystems). The PCR thermal cycling was programmed as follows: 95°C for 15 min followed by 40 cycles of 94°C for 30 s, 55°C for 90 s, 72°C for 60 s, and a final cycle at 72°C for 10 min. One microliter of 10 times dilution PCR product was mixed with 8.5 µL of Hi-Di formamide and 0.125 µL of ROX size standard (Applied Biosystems). The mixture was denatured at 95°C for 5 min and then run on an ABI PRISM 3130xl DNA Analyzer (Applied Biosystems). Allele size for the highest stutter peak with the height ranging between 500 and 10,000 RFU were recorded. The genotyping was performed using GeneMapper 4.0 (Applied Biosystems).

Thirty-eight EST-SSR primer pairs amplified one product in diploid species and two discrete products in tetraploid species with height ranging RFU (Table S2) were selected for further analysis in the 268 *Vigna* accessions. Three primers with different labels and size products were mixed into a single PCR reaction mixture and amplified as multiplex PCR. After genotyping, a single allele size was scored for each marker in each accession corresponding to the strongest peak.

### Data Analysis

Since both diploid (1 genome set) and tetraploid (2 genome set) species were analyzed together in this study and each SSR primer pair detected 2 loci in the tetraploid species, we separated the genome of *V. reflexo-pilosa*, into 2 different diploid types, viz. A and B. Therefore 10 genomes were recognized from the 8 taxa of 7 *Vigna* species.

Genetic distance (*D*_A_) [24] for all possible pairs of accessions was calculated using software POPULATIONS 1.2.28 (available at www.cnrs-gif.fr/pge/bioinfo/populations). *D*_A_ among the 10 genomes was also calculated using the same software. A phylogenic tree was constructed to reveal relationships among accessions based on *D*_A_ using neighbor-joining clustering method by software MEGA 5.05 [25] with bootstrap support (1,000 replicates) obtained by re-sampling the allelic frequency data. In order to confirm the results from neighbor-joining clustering, principal coordinate analysis (PCoA) was also performed using software PAST [26] to reveal the relationship among different accessions. In addition, based on sources of *V. hirtella*, Seehalak et al. (2006) divided *V. hirtella* in to 3 subgroups (a1, a2 and b), thus the relationship of individuals based on PCoA analysis enabled 12 sub-genome groups to be determined. Using average *D*_A_ genetic distance between 12 sub-genomes, an unrooted dendrogram showing relationships between these genomes was constructed by neighbor-joining method using software POPULATIONS 1.2.32. (available at www.cnrs-gif.fr/pge/bioinfo/populations) with bootstrap support (400 replicates) obtained by re-sampling the allelic frequency data. The trees were visualized with MEGA ver. 5.05 [25].

## Results

### Azuki Bean EST-SSR Markers

A total of 11,167 cDNA clones were sequenced consisting of 7,534 clones from a seedling library and 3,633 clones from a young pod library. After clustering, 4,896 potential non-redundant EST sequences, including 2,350 contigs and 2,546 singletons, were generated with a total of 4,284,693 qualified bases (Table S3). By using the fuzznuc program in EMBOSS, a total of 1,188 SSRs were identified in the 4,896 non-redundant EST sequences. Of the 1,188 SSRs, di-, tri-, and tetra-nucleotide SSRs accounted for 71.6%, 26.7%, and 1.7%, respectively (Table S4). Assuming that total length of the non-redundant azuki bean EST sequences is 4.3 Mbp, the frequency of occurrence of the SSRs in transcribed regions was estimated to be one in every 3.6 kb. Altogether105 primer pairs were initially designed on the flanking regions of the 132 perfect SSR motifs (Table S4). To increase the number of candidate EST-SSR markers, additional 1,324 primer pairs were designed on the flanking regions of 1,545 imperfect SSR motifs allowing one or two base mismatching. As a result, a total of 1,429 EST-SSR markers were designed (Table S4), of which 149, 28, 5 and 5 markers identified 2, 3, 4 and ≤5 SSRs in the regions between the primer pairs, respectively. Thus the total number of identified SSRs by the 1,429 EST-SSR markers was 1,677 that consisted of 137 (8.2%) di-nucleotide repeats, 1,400 (83.5%) tri-nucleotide repeats and 140 (8.3%) tetra-nucleotide repeats (Table S4). Among the di-nucleotide repeats, poly(AG)_n_ (n = 88, 5.2% of total) were most frequently observed, followed by poly(AT)_n_ (n = 35, 2.1%) and poly (AC)_n_ (n = 14, 0.8%). Among the ten types of tri-nucleotide repeats observed, poly(AAG)_n_ (n = 322, 19.2%) were the most abundant, followed by poly(GGA)_n_ (n = 195, 11.6%) and poly(ATC)_n_ (n = 180, 10.7%). Among the thirteen tetra-nucleotide repeats, poly(AAAG)_n_ (n = 45, 2.7%) were the most frequently observed, followed by poly(AAAT)_n_ (n = 30, 1.8%), and poly(AAAC)_n_ (n = 20, 1.2%). The details of the designed azuki bean EST-SSR primers, along with the corresponding SSR motif, product size, and primer sequence, are available at http://marker.kazusa.or.jp/Azuki and in Table S5.

### Amplification and Transferability of Azuki Bean EST-SSR Markers

Transferability and polymorphism of the 1,429 azuki bean EST-SSR markers were initially examined by polyacrylamide gel electrophoresis using eight accessions consisting of four major *Vigna* crop species; azuki bean complex, black gram, rice bean and mungbean (Table 1, Table S1). These wild and cultivated parental accessions had been used for a linkage map construction in each species ([27] [28] [29] [30]. Amplification of azuki bean EST-SSR markers in the same species, *V. angularis*, and the closely related species, *V. umbellata,* were 91.3 and 92.9% which were higher than those in *V. radiata* and *V. mungo* (Table 1). However, the size and pattern of PCR products of EST-SSR markers did not always reveal simple banding pattern with expected size, even in the same species. Among the amplified markers, 81.8 to 83.6% of them revealed simple banding pattern suitable for further application. When polymorphism between cultivated and wild accessions was examined, *V. radiata* possessed the highest polymorphism in which 277 markers (23.1% of amplified markers) were polymorphic. Further, 175 markers with good transferability in the four *Vigna* species were tested for polymorphism in a panel of 16 accessions from 8 taxa of Asian *Vigna* (Table S1). The test revealed that 22 markers (12.6%) failed to amplify, 36 (20.6%) amplified some accessions, whereas 117 (66.8%) successfully amplified all the 16 accessions (Table 2). Among the 117 amplifiable markers, 2 (1.1%) gave product size larger than 500 bp, 4 (2.3%) amplified multiple products, 45 (25.7%) amplified one product in tetraploid species, 10 (5.7%) were monomorphic, and 56 (32%) were polymorphic, amplifying one product in diploid species and two discrete products in tetraploid species and were considered to be suitable for analyzing origin of genome donor of the tetraploid species.

**Table 2:** Characteristics of 196 EST-SSR primers developed in this study.

Type	Description		Number of primers (%)
1	Not amplified in all accessions		**22 (12.6)**
	Amplified in some accessions		**36 (20.6)**
2–1)		1) Amplified in 1 to 8 accession(s)	5 (2.9)
2–2)		2) Amplified in 9 to 15 accessions	31 (17.7)
	Amplified in all 16 accessions		**117 (66.8)**
3–1)		1) PCR product of more than 500 bp	2 (1.1)
3–2)		2) Multiple PCR product	4 (2.3)
3–3)		3) Monomorphic PCR product	10 (5.7)
3–4)		4) Single PCR product in tetraploid	45 (25.7)
3–5)		5) Two PCR products in tetraploid	56 (32)
Total			**175 (100.0)**

### EST-SSR Polymorphism and Genetic Diversity

Thirty-eight primer pairs of azuki bean EST-SSR were analyzed in 268 accessions of 10 genomes from eight taxa of Asian *Vigna*. Based on discrimination of the diploid species we distinguished two discrete products detected by each marker of *V. reflexo-pilosa* into two types, viz. A and B. The product size that is the same or very similar to *V. trinervia* was scored as type A, while the other product size was scored as type B. In total, 445 alleles were detected in 10 genomes by the 38 EST-SSR loci (Tables 3, 4). The number of alleles detected per locus ranged between 3 (VES0777 and VES1271) and 34 (VES1172) with a mean of 11.7 alleles per locus (Table 4). The *PIC* values ranged from 0.36 (VES0116) to 0.94 (VES1172) with a mean of 0.67. None of the EST-SSR markers had *PIC* values lower than 0.3, while 20 markers had *PIC* values higher than 0.7. The markers showed high *PIC* value in *V. hirtella* (0.44) and *V. minima* (0.42), but low value in *V. reflexo-pilosa* (0.04-0.09). In general, each marker showed a *PIC* value of 0 in both populations of *V. reflexo-pilosa*. The allelic richness was between 2.0 for marker VES0777 and 9.9 for marker VES1172 with a mean of 4.7. Fifteen primers had allelic richness higher than 5.0 (Table 4).

**Table 3:** Genome number assigned in this study with number of alleles, gene diversity and observed heterozygosity analyzed by 38 EST-SSR markers.

Genome	No. of accessions	No. of loci typed	No. of alleles	Gene diversity	Observed heterozygosity
1 (*Vigna exilis*)	13	38	100	0.305	0.012
2 [*V. reflexo-pilosa* var. *glabra* (a)]	7	38	44	0.053	0.000
3 [*V. reflexo-pilosa* var. *glabra* (b)]	7	38	44	0.052	0.000
4 (*V. hirtella*)	47	38	171	0.478	0.019
5 (*V. minima*)	49	38	203	0.458	0.017
6 [*V. reflexo-pilosa* var. *reflexo-pilosa* (a)]	51	38	56	0.058	0.000
7 [*V. reflexo-pilosa* var. *reflexo-pilosa* (b)]	51	38	76	0.094	0.000
8 (*V. radiata* var. *sublobata*)	13	38	101	0.357	0.008
9 (*V. tenuicaulis*)	42	38	122	0.309	0.022
10 (*V. trinervia*)	46	38	98	0.306	0.028
Total	326	38	445	0.711	0.013

**Table 4:** EST-SSR primers used, number of alleles per locus, allele size range, polymorphic information content (PIC) and allelic richness for each genome.

			PIC											Allelic richness										
			Genome number											Genome number										
Primer	No. of alleles	Allele size range (bp)†	1	2	3	4	5	6	7	8	9	10	Overall	1	2	3	4	5	6	7	8	9	10	Overall
VES0019	16	253–283 (30)	0.45	0.00	0.21	0.77	0.81	0.00	0.07	0.40	0.85	0.61	0.77	3.6	1.0	2.0	5.9	6.2	1.0	1.5	2.8	7.2	3.6	5.8
VES0021	18	217–289 (72)	0.46	0.00	0.00	0.51	0.59	0.00	0.00	0.13	0.37	0.04	0.75	3.0	1.0	1.0	3.6	4.1	1.0	1.0	1.8	3.0	1.3	5.3
VES0070	7	265–275 (10)	0.00	0.00	0.00	0.50	0.46	0.00	0.07	0.64	0.17	0.00	0.61	1.0	1.0	1.0	3.3	2.7	1.0	1.5	3.8	2.0	1.0	3.5
VES0093	11	183–199 (16)	0.00	0.00	0.00	0.51	0.15	0.00	0.00	0.66	0.05	0.40	0.75	1.0	1.0	1.0	3.3	1.9	1.0	1.0	3.9	1.3	2.3	5.0
VES0116	4	278–284 (6)	0.13	0.37	0.00	0.00	0.00	0.13	0.00	0.13	0.00	0.22	0.36	1.8	2.0	1.0	1.0	1.0	1.7	1.0	1.8	1.0	1.9	2.2
VES0120	16	245–293 (48)	0.13	0.00	0.00	0.16	0.52	0.00	0.10	0.73	0.00	0.00	0.51	1.8	1.0	1.0	2.0	4.2	1.0	1.6	4.8	1.0	1.0	3.5
VES0202	15	218–254 (36)	0.55	0.00	0.00	0.58	0.24	0.00	0.00	0.43	0.46	0.00	0.71	4.4	1.0	1.0	3.4	2.1	1.0	1.0	2.9	2.9	1.0	4.6
VES0204	20	310–366 (56)	0.61	0.00	0.00	0.69	0.70	0.10	0.24	0.44	0.21	0.31	0.71	4.5	1.0	1.0	4.7	5.4	1.6	2.6	2.8	2.4	2.5	5.0
VES0335	15	265–284 (19)	0.44	0.00	0.21	0.78	0.29	0.04	0.40	0.00	0.60	0.55	0.81	2.8	1.0	2.0	6.4	2.3	1.3	2.7	1.0	3.7	3.6	6.5
VES0427	9	316–329 (13)	0.50	0.32	0.00	0.26	0.47	0.00	0.00	0.34	0.16	0.31	0.71	3.0	2.0	1.0	2.1	3.3	1.0	1.0	2.8	1.8	2.4	4.5
VES0478	11	298–309 (11)	0.72	0.00	0.00	0.22	0.21	0.13	0.13	0.50	0.09	0.08	0.66	5.4	1.0	1.0	1.9	1.9	1.7	1.7	3.3	1.6	1.5	4.3
VES0546	26	431–476 (45)	0.63	0.00	0.00	0.32	0.84	0.00	0.37	0.48	0.58	0.48	0.85	4.4	1.0	1.0	2.9	7.2	1.0	3.2	3.0	3.0	2.9	7.5
VES0624	4	265–271 (6)	0.00	0.00	0.00	0.00	0.15	0.04	0.00	0.00	0.00	0.00	0.42	1.0	1.0	1.0	1.0	1.9	1.3	1.0	1.0	1.0	1.0	2.6
VES0665	9	195–213 (18)	0.26	0.00	0.00	0.31	0.35	0.00	0.00	0.67	0.13	0.24	0.68	2.6	1.0	1.0	2.7	2.8	1.0	1.0	5.0	1.9	2.3	4.2
VES0670	6	99–114 (15)	0.44	0.00	0.00	0.56	0.20	0.00	0.00	0.00	0.38	0.08	0.71	2.8	1.0	1.0	3.8	2.1	1.0	1.0	1.0	3.1	1.5	4.4
VES0678	7	293–318 (25)	0.45	0.00	0.00	0.40	0.54	0.00	0.00	0.36	0.28	0.41	0.70	2.9	1.0	1.0	2.3	3.3	1.0	1.0	2.0	2.0	2.5	4.5
VES0679	18	310–365 (55)	0.44	0.00	0.00	0.40	0.78	0.31	0.14	0.63	0.61	0.74	0.81	2.8	1.0	1.0	3.2	5.6	2.0	1.9	4.4	3.9	5.2	7.8
VES0749	15	208–236 (28)	0.29	0.00	0.00	0.60	0.27	0.00	0.00	0.62	0.09	0.43	0.79	2.0	1.0	1.0	3.8	2.3	1.0	1.0	4.4	1.6	2.5	5.8
VES0762	7	242–266 (24)	0.13	0.00	0.21	0.62	0.08	0.00	0.04	0.37	0.00	0.00	0.43	1.8	1.0	2.0	3.6	1.5	1.0	1.3	2.0	1.0	1.0	3.0
VES0777	3	166–175 (9)	0.00	0.00	0.00	0.36	0.40	0.00	0.10	0.18	0.14	0.00	0.37	1.0	1.0	1.0	2.0	2.3	1.0	1.6	1.9	1.7	1.0	2.0
VES0803	12	292–308 (16)	0.00	0.00	0.00	0.64	0.71	0.00	0.10	0.29	0.41	0.00	0.80	1.0	1.0	1.0	4.8	4.6	1.0	1.6	2.0	2.6	1.0	6.3
VES0868	12	213–288 (75)	0.13	0.00	0.00	0.53	0.27	0.00	0.00	0.26	0.35	0.00	0.62	1.8	1.0	1.0	4.1	2.2	1.0	1.0	2.6	2.0	1.0	4.2
VES0987	9	298–310 (12)	0.26	0.21	0.00	0.55	0.36	0.00	0.00	0.13	0.31	0.59	0.73	2.6	2.0	1.0	3.4	3.0	1.0	1.0	1.8	2.0	3.0	5.0
VES1001	10	227–250 (23)	0.29	0.00	0.00	0.39	0.55	0.00	0.00	0.64	0.00	0.00	0.65	2.5	1.0	1.0	2.7	3.6	1.0	1.0	3.9	1.0	1.0	3.9
VES1020	6	170–183 (13)	0.26	0.00	0.00	0.48	0.51	0.07	0.07	0.00	0.00	0.37	0.73	2.6	1.0	1.0	2.9	3.3	1.5	1.5	1.0	1.0	2.0	4.6
VES1023	7	140–157 (17)	0.00	0.00	0.00	0.28	0.56	0.00	0.10	0.00	0.37	0.00	0.59	1.0	1.0	1.0	2.2	3.7	1.0	1.6	1.0	2.2	1.0	3.4
VES1029	9	111–128 (17)	0.58	0.00	0.00	0.62	0.04	0.00	0.00	0.00	0.52	0.00	0.61	3.8	1.0	1.0	3.8	1.3	1.0	1.0	1.0	2.9	1.0	3.9
VES1067	14	447–477 (30)	0.13	0.00	0.37	0.68	0.60	0.00	0.32	0.36	0.17	0.37	0.83	1.8	1.0	2.0	4.5	3.9	1.0	2.8	2.0	2.1	2.0	6.7
VES1082	14	281–317 (36)	0.40	0.00	0.00	0.51	0.70	0.00	0.00	0.34	0.13	0.46	0.70	2.8	1.0	1.0	3.1	4.8	1.0	1.0	2.8	1.9	2.8	5.0
VES1085	13	390–403 (13)	0.00	0.00	0.00	0.33	0.60	0.31	0.13	0.00	0.50	0.74	0.82	1.0	1.0	1.0	2.6	4.1	2.5	1.7	1.0	2.8	4.7	6.6
VES1172	34	217–268 (51)	0.71	0.53	0.37	0.85	0.89	0.58	0.82	0.60	0.41	0.59	0.94	6.0	3.0	2.0	7.4	9.0	3.8	6.5	3.8	2.9	4.3	9.9
VES1196	5	203–215 (12)	0.00	0.00	0.00	0.30	0.15	0.00	0.00	0.00	0.09	0.00	0.38	1.0	1.0	1.0	2.5	1.9	1.0	1.0	1.0	1.5	1.0	2.1
VES1231	6	120–139 (19)	0.23	0.00	0.00	0.53	0.08	0.00	0.00	0.00	0.34	0.00	0.52	2.0	1.0	1.0	2.9	1.5	1.0	1.0	1.0	2.6	1.0	3.5
VES1258	10	369–398 (29)	0.54	0.00	0.00	0.22	0.59	0.04	0.00	0.00	0.50	0.48	0.76	3.0	1.0	1.0	1.9	3.6	1.3	1.0	1.0	2.9	3.0	5.5
VES1263	8	305–320 (15)	0.13	0.00	0.00	0.00	0.43	0.07	0.00	0.56	0.00	0.30	0.68	1.8	1.0	1.0	1.0	2.7	1.5	1.0	3.6	1.0	2.0	4.4
VES1271	3	307–310 (3)	0.00	0.00	0.00	0.00	0.00	0.00	0.00	0.00	0.05	0.37	0.42	1.0	1.0	1.0	1.0	1.0	1.0	1.0	1.0	1.3	2.0	2.6
VES1310	7	288–309 (21)	0.23	0.00	0.00	0.37	0.25	0.00	0.10	0.60	0.60	0.30	0.55	2.0	1.0	1.0	3.0	2.5	1.0	1.6	3.8	3.6	2.0	3.3
VES1469	29	128–178 (50)	0.00	0.21	0.21	0.82	0.79	0.17	0.04	0.61	0.64	0.66	0.86	1.0	2.0	2.0	6.5	6.1	2.0	1.3	3.8	5.3	5.0	7.7
Total	445																							
Average	11.7		0.28	0.04	0.04	0.44	0.42	0.05	0.09	0.32	0.28	0.27	0.67	2.43	1.16	1.16	3.24	3.34	1.27	1.53	2.49	2.33	2.15	4.75

† Difference between the largest and smallest fragments amplified by each primer is shown in parentheses.

See Table 3 for the abbreviations of genome number.

*V. hirtella* possessed the highest gene diversity (0.478), while *V. reflexo-pilosa* showed very low gene diversity (0.053 to 0.094) (Table 3). All genomes of *V. reflexo-pilosa* showed no observed heterozygosity, while the other genomes showed very low observed heterozygosity from 0.008 to 0.028 (Table 3).

### Genetic Relationship among Genomes

Genetic distance (*D*_A_) among the ten genomes is shown in Table 5a. In most cases *D*_A_ among genomes was high (>0.6). Nonetheless, *D*_A_ between *V. trinervia* and *V. reflexo-pilosa* var. *glabra* (A) or *V. reflexo-pilosa* var. *reflexo-pilosa* (A) was low being 0.333 and 0.313, respectively. While, both *V. reflexo-pilosa* var. *glabra* (B) and *V. reflexo-pilosa* var. *reflexo-pilosa* (B) showed lowest *D*_A_ with *V. hirtella* being 0.585 and 0.589 (Table 5a) or with *V. hirtella* (a1), being 0.429 and 0.422 (Table 6), respectively. Interestingly, *V. trinervia*, which belongs to section *Angulares* showed lower *D*_A_ with *V. radiata* var. *sublobata* (section *Ceratotropis*) than with those species in the section *Angulares*.

**Table 5:** Genetic distance(*D*_A_) within and among 10 genomes with (*Vigna hirtella* is not divided).

Genome	1	2	3	4	5	6	7	8	9	10
1	**0.326**									
2	0.900	**0.061**								
3	0.695	0.985	**0.060**							
4	0.649	0.927	0.585	**0.482**						
5	0.740	0.881	0.740	0.693	**0.464**					
6	0.901	0.079	0.987	0.922	0.870	**0.059**				
7	0.690	0.989	0.098	0.589	0.740	0.990	**0.097**			
8	0.848	0.817	0.875	0.838	0.884	0.817	0.878	**0.385**		
9	0.712	0.895	0.716	0.652	0.716	0.894	0.720	0.829	**0.310**	
10	0.891	0.333	0.963	0.890	0.886	0.313	0.965	0.799	0.849	**0.304**

**Table 6:** Genetic distance(*D*_A_) within and among 10 genomes (*Vigna hirtella* is divided into three genomes).

Genome	1	2	3	4 (a1)	4 (a2)	4 (b)	5	6	7	8	9	10
1	**0.326**											
2	0.900	**0.061**										
3	0.695	0.985	**0.060**									
4 (a1)	0.625	0.911	0.429	**0.377**								
4 (a2)	0.626	0.931	0.600	0.609	**0.298**							
4 (b)	0.749	0.926	0.672	0.672	0.646	**0.321**						
5	0.740	0.881	0.740	0.704	0.689	0.700	**0.464**					
6	0.901	0.079	0.987	0.908	0.925	0.922	0.870	**0.059**				
7	0.690	0.989	0.098	0.422	0.606	0.680	0.740	0.990	**0.097**			
8	0.848	0.817	0.875	0.899	0.824	0.831	0.884	0.817	0.878	**0.385**		
9	0.712	0.895	0.716	0.713	0.697	0.446	0.716	0.894	0.720	0.829	**0.310**	
10	0.891	0.333	0.963	0.892	0.895	0.874	0.886	0.313	0.965	0.799	0.849	**0.304**

See Table 3 for the abbreviations of genome number.

Genetic distances within and among genomes are shown on the diagonal and below the diagonal, respectively.

The accessions that belong to genome a1, a2 and b are shown in Figure 3.

**Figure 2:**
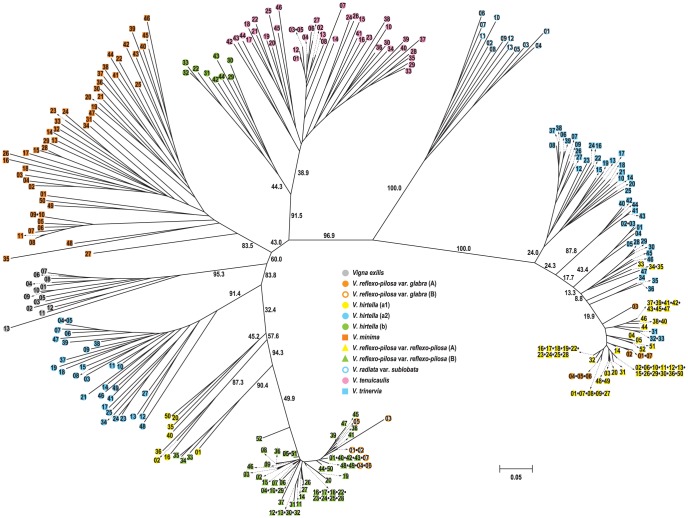
A phylogenetic tree showing relationship among 286 accessions in 12 sub-genome groups of the genus *Vigna* subgenus *Ceratotropis* based on variation at 38 EST-SSR loci.

**Figure 3:**
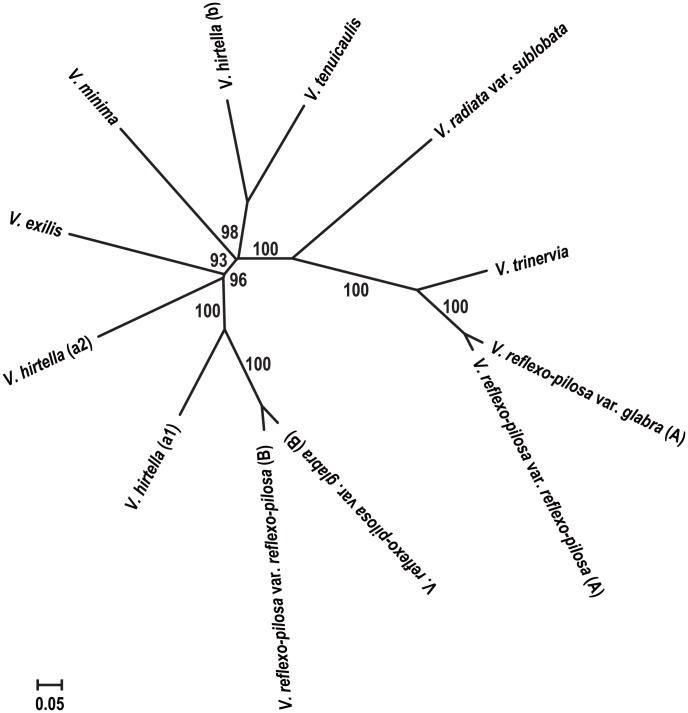
A phylogenetic tree showing relationship among 12 sub-genome groups of the genus *Vigna* subgenus *Ceratotropis* constructed based on the genetic distance shown in Table 6.

A neighbor-joining tree was constructed based on the genetic distances for all possible pairs of 268 accessions. The tree showed two major groups with 96.9% bootstrap value, namely azuki bean group and mungbean group (Figure 2). *V. exilis*, *V. minima*, *V. tenuicaulis*, *V. hirtella*, *V. reflexo-pilosa* var. *glabra* (B), and *V. reflexo-pilosa* var. *reflexo-pilosa* (B) were clustered in the azuki bean group. While *V. radiata* var. *sublobata*, *V. trinervia*, *V. reflexo-pilosa* var. *glabra* (A) and *V. reflexo-pilosa* var. *reflexo-pilosa* (A) were clustered in the mungbean group. In the azuki bean group, 13 accessions of *V. exilis* and 49 accessions of *V. minima* were independent clusters with 95.3% and 83.5% bootstrap values, respectively. *V. hirtella* showed high divergence in which 9 accessions in the population of *V. hirtella* (b) clustered strongly with *V. tenuicaulis* with 91.5% bootstrap value, while the other 38 accessions in *V. hirtella* (a) clustered with *V. reflexo-pilosa* var. *glabra* (B) and *V. reflexo-pilosa* var. *reflexo-pilosa* (B) with 83.8% bootstrap value. Those 38 accessions of *V. hirtella* also showed two subclusters a1 and a2 (Figure 2 and 3). In the mungbean group, *V. trinervia* clustered with *V. reflexo-pilosa* var. *glabra* (A) and *V. reflexo-pilosa* var. *reflexo-pilosa* (A) with a 100% bootstrap support. Five accessions from Myanmar (40–44) formed a distinct branch with a bootstrap value of 87.8% and were separated from the other accessions (Figure 2). Three accessions from Laos (31–33) were differentiated from the other accessions and were clustered together with *V. reflexo-pilosa* (A) accessions.

**Figure 4:**
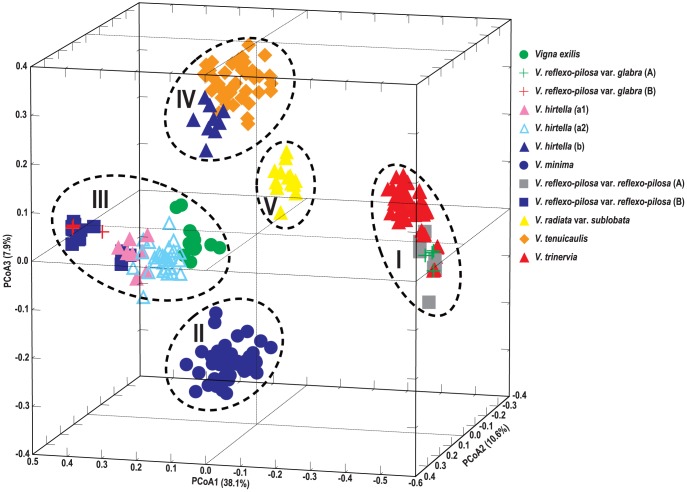
PCA scattered plot depicting relationship among 286 accessions in 12 sub-genome groups of the genus *Vigna* subgenus *Ceratotropis* based on variation at 38 EST-SSR loci.

The principle coordinate analysis (PCoA) was also conducted to confirm species relationship based on the neighbor-joining tree using the same genetic distance estimates. The first three PCs together accounted up to 56.6% of the total variation. The first, second and third PCs accounted for 38.1%, 10.6% and 7.9%, respectively (Figure 4). A three-dimensional PC plot (PC1, PC2 and PC3) of the twelve genome groups is shown in Figure 4, which clearly separated the genomes into 5 distinct groups. Group I comprised *V. reflexo-pilosa* var. *reflexo-pilosa* (A), *V. reflexo-pilosa* var. *glabra* (A), and *V. trinervia*. Group II comprised *V. minima* only. Group III comprised *V. reflexo-pilosa* var. *glabra* (B), *V. reflexo-pilosa* var. *reflexo-pilosa* (B), *V. exilis* and *V. hirtella* (a1, a2). Group IV comprised *V. hirtella* (b), and *V. tenuicaulis*. Group V comprised solely *V. radiata* var. *sublobata*. Distribution range of *V. reflexo-pilosa* var. *glabra* (A) and *V. reflexo-pilosa* var. *reflexo-pilosa* (A) in Group I was narrower than *V. reflexo-pilosa* var. *glabra* (B) and *V. reflexo-pilosa* var. *reflexo-pilosa* (B) in Group III. Of all species used in this study, only *V. hirtella* was unambiguously distinguished into two sub-groups, a and b. Subgroup a showed close relationship with populations of *V. reflexo-pilosa* var. *reflexo-pilosa* (B); *V. reflexo-pilosa* var. *glabra* (B) and *V. exilis*, while subgroup b showed close relationship with *V. tenuicaulis* (Figure 3).

## Discussion

### Transferability of Azuki Bean EST-SSRs

One of the advantages of SSRs derived from EST (EST-SSR markers) to other DNA marker systems is the high transferability across species and genera because of primer pairs are designed from conserved transcribed regions, although they are generally less polymorphic than genomic SSR markers (gSSR). In this study, more than 83% of the azuki bean EST-SSR markers were able to amplify DNA from 8 Asian *Vigna* species including four major crop species, viz. *V. angularis*, *V. umbellata, V. radiata* and *V. mungo* of the subgenus *Ceratotropis*. This result is in agreement with that of [31] who reported that more than 80% of mungbean EST-SSR markers were transferable to other species in the subgenus *Ceratotropis*. Transferability of the EST-SSR markers from azuki bean (in this study) and mungbean [31] is greater than that of gSSR markers from azuki bean (>67% transferable; [28]) and mungbean (>59% transferable; [32]). The high transferability of azuki bean EST-SSRs and of mungbean EST-SSRs to the related *Vigna* species indicates high genome conservation among the species in the subgenus *Ceratotropis*. Therefore, the azuki bean EST-SSR markers developed in this study will be useful for comparative genomic and genetic diversity studies in *Vigna* species. Nonetheless, as expected, polymorphism rate of the azuki bean EST-SSR markers detected between cultivated and wild accessions of azuki bean, rice bean, mungbean and black gram is low (14% in black gram and 23% in mungbean). In the same plant materials, Chaitieng et al. [28] reported that polymorphism rate of the genomic azuki bean SSRs was as high as 50% in black gram to 63% in mungbean. Thus low polymorphism rate of the azuki bean EST-SSRs can be an undesirable character for the use of these markers in comparative genome mapping in the genus *Vigna*. However, EST-SSRs represent true genetic diversity which may be more directly associated with traits of interest in breeding as compared to gSSRs [33], and they may reflect better relationships among the related species in genetic diversity study.

### Evolution and Domestication of Tetraploid *V. reflexo-pilosa*

*V. reflexo-pilosa* is the only tetraploid species of the genus *Vigna*. Although previous studies on *Vigna* species using DNA markers and plastid sequences clearly demonstrated that *V. trinervia* is a genome donor of *V. reflexo-pilosa*, those studies were unable to identify the other genome donor. In the present study, five putative genome donor species of *V. reflexo-pilosa* belong to the section *Angulares* and one species representing the section *Ceratotropis* were analyzed with azuki bean EST-SSRs. By assigning the discrete PCR products of *V. reflexo-pilosa* into two distinguished genomes (designated as genome A and B), we were able to identify the two genome donor parents of créole bean. Phylogenetic tree (Figure 2) and PCoA plot (Figure 4) clearly demonstrated that genome A of *V. reflexo-pilosa* is received from *V. trinervia*. This result confirms the previous results reported by [7] [10] [13] [14] [15] that *V*. *trinervia* is a diploid genome donor. *V*. *trinervia* and *V. reflexo-pilosa* shares several similar morphological characters such as seed shape (rectangular), non-protruded hilum and large golden flower. The phylogenetic tree and the PCoA plot also unambiguously demonstrated that the other genome (genome B) of *V. reflexo-pilosa* is originated from *V. hirtella*. This result is in agreement with the result reported by Tateishi [7] [10] [13] [16] that *V. hirtella* is one of candidate genome donor of créole bean. Results from phylogenetic analysis of plastid DNA sequences of [14] [15] suggested that *V. trinervia* is the maternal genome donor of *V. reflexo-pilosa.* Since the previous and our results demonstrated close genetic relationship between tetraploid *V. reflexo-pilosa* and diploid *V. trinervia*/*V. hirtella*, we propose that créole bean evolved from interspecific hybridization between *V. trinervia* as female parent and *V. hirtella* as male parent, followed by genome duplication.

Phylogenetic tree (Figure 2) and PCoA plot (Figure 4) showed lower divergence of genome A of *V. reflexo-pilosa* compared to genome B. Very low gene diversity within both genomes A and B suggested that this tetraploid species has a monophyletic (single) origin and only evolved recently. Gene diversity within the genome A in both *V. reflexo-pilosa* var. *reflexo-pilosa* (wild form) and *V. reflexo-pilosa* var. *glabra* (cultivated form) is very similar (0.058 vs. 0.053), while gene diversity of the genome B in wild créole bean is about twice of that in the cultivated one (0.94 vs. 0.52) (Table 3). This indicates that the evolution rate of EST-SSRs on genome B of créole bean might be faster than that on genome A. The marked morphological differences between the cultivated and wild créole beans are thicker and erect glabrous stem in the former. Moreover, cultivated *V. reflexo-pilosa* still possesses a relatively high degree of pod shattering (Somta and Chankaew, personal observation), an important domestication trait for legume crops [34]. Therefore, the cultivated *V. reflexo-pilosa* can be treated as a semi-domesticated form. Based on cultivation, utilization and distribution of diploid genome donor species, créole bean appears to have been domesticated in Southeast Asia. The crop is now very rarely cultivated, although it is produced in northern mountainous villages in Vietnam under the same name and consumed in the same way as mungbean [2].

### Genetic Structure of Diploid Asian *Vigna* Species Detected by EST-SSR

We found that *V. hirtella* possessed greater genetic variation than *V. exilis*, *V. minima*, and *V. tenuicaulis*. A previous study based on AFLP suggested that *V. hirtella* germplasm collected from Thailand, Malaysia and Myammar is a species complex consisting of two taxa (types), called *V. hirtella* (a) and *V. hirtella* (b) [35]. Although the study used only 3 accessions for *V. hirtella* (a) and 5 accessions for *V. hirtella* (b), all the 3 *V. hirtella* (a) accessions were included in *V. hirtella* (a1) group, and 5 *V. hirtella* (b) accessions were included in *V. hirtella* (a2) group in the present study. In addition, our EST-SSR results detected the existence of another genetically distinct type of materials designated as *V. hirtella* (b) which is closely related to *V. tenuicaulis* (Figure 2, 3, 4). These accessions should be re-examined morphologically whether they can be included within a morphological variation of *V. tenuicaulis.*

*V. trinervia* is widely distributed across Asia and also found in Papua New Guinea, Madagascar and East Africa. It is the second most widely distributed species in the subgenus *Ceratotropis* after *V. radiata* var. *sublobata* [2]. Nevertheless, gene diversity of *V. trinervia* was not high as compared to the other related species (Table 3). In Southeast Asia, especially in Thailand and the peninsular Malaysia, *V. trinervia* is often found on rural roadside habitats and in or near to rubber or oil palm plantations. It seems that some of the populations in those areas were introduced recently as a cover plant in the plantations [2]. This may account for the low diversity of *V. trinervia.*

*V. tenuicaulis* distributes in open wet habitats in northern Southeast Asia, and appeared to be closely related to *V. hirtella* (b) and *V. angularis* [13] [35] [6] [36]. Tomooka et al. suggested that there are no major barriers to hybridization between *V. tenuicaulis* and *V. hirtella* (b) [2]. Our results confirmed the close relationship between these two species (Figure 2, 4). Previous studies based on molecular, biochemical and morphological variations all showed high level of distinctness and variation within *V. tenuicaulis*. EST-SSR marker variation in our study revealed contrasting results. Although we used as many as 30 accessions of *V. tenuicaulis* from three countries, viz. Laos, Myanmar and Thailand. (Figure 1, Table S1), the species showed relatively low gene diversity (0.309).

*V. exilis* has been reported only in Thailand and Myanmar [2] [14]. In Thailand, this species is restricted to rocky limestone mountains. Its habitats suggest that it may be useful as gene sources for resistant to alkaline soil and drought conditions. SSR analysis revealed high level of intra-specific diversity in *V. exilis* [37]. Our results also supported this despite only 13 accessions from narrow geographical origin (west of Thailand) of this species were used (Figure 1, Table S1), the species showed similar level of gene diversity to *V. tenuicaulis* and *V. trinervia* (Table 3).

*V. minima* has broad environments adaptation and grows well in shaded deciduous forest floors and open-wet habitats in East and Southeast Asia [2]. It is the only species in section *Angulares* that is found on the forest floor. Among the Asian *Vigna* species analyzed in this study, *V. minima* is the second most diverse species after *V. hirtella* (Table 3). The high differentiation of *V. minima* is due to wide geographical distribution of the analyzed accessions. In Southeast Asia, isolation of *V. minima* in forests in different mountainous regions across Thailand and Myanmar and its sporadic occurrence in patches of forests in those regions may account for high level of population divergence [35]. Accessions of *V. minima* in the present study were most closely related with *V. hirtella*, especially *V. hirtella* (b), followed by *V. exilis* (Figure 2, 4). Similar finding was reported by [35]. This agrees with their morphological appearance [2] that *V. minima* is sometimes confused with *V. hirtella. V. minima* can be distinguished from *V. hirtella* by smaller bracteole and more protruding hilum with well-developed rim-aril [2].

In summary, one of the advantages of EST-SSR markers to other DNA marker systems is their co-dominant nature and high transferability to closely related species. In this study, we used EST-SSR primer pairs, which amplified one PCR product in diploid species and two discrete PCR products in tetraploid species to separate genomes of *V. reflexo-pilosa* into 2 different diploid types, viz. A and B, that could successfully determine different sets of the donor genomes. Phylogenetic analyses revealed that *V. trinervia* and *V. hirtella* are the genome donors of *V. reflexo-pilosa.* Both genomes of cultivated and wild créole bean accessions showed low divergence suggesting that domestication of *V. reflexo-pilosa* is a relatively recent event.

## Supporting Information

Table S1 **Origin of 268 accessions used to analyze origin of genome of** ***V. reflexo-pilosa*** **.**
(XLSX)

Table S2 **Transferability and polymorphisms of azuki bean EST-SSR markers in the other** ***Vigna*** **species.**
(XLSX)

Table S3 **EST composition in non-redundant azuki bean EST sequences.**
(XLSX)

Table S4 **Summary of SSRs in the non-redundant azuki bean ESTs and designed EST-SSR primers.**
(XLSX)

Table S5 **Primer sequences, SSR motifs, expected sizes and EST sequences of the designed EST-SSR markers.**
(XLSX)
